# Water Desalination Using the Once-through Multi-Stage Flash Concept: Design and Modeling

**DOI:** 10.3390/ma15176131

**Published:** 2022-09-03

**Authors:** Qahtan Thabit, Abdallah Nassour, Michael Nelles

**Affiliations:** 1 Department of Waste and Resource Management, Faculty of Agricultural and Environmental Sciences, University of Rostock, D-18059 Rostock, Germany; 2 Deutsches Biomasseforschungszentrum GmbH, D-04347 Leipzig, Germany

**Keywords:** thermal energy, water desalination, once-through mult-istage flash, water scarcity, optimization

## Abstract

Thermal water desalination is one of the most important techniques to solve the water scarcity problem in many regions of the world. Out of around 7.8 billion people in the world, only about 6 billion of them have access to clean water; notably, climate change plays a major role in accelerating the evaporation rate of water from water bodies, which in turn increases the scarcity. Multi-stage flash, recognized to have a high rate of water production in comparison with other available technologies, accounts for 35% of water desalination facilities worldwide. This paper presents a detailed Excel model to evaluate the amount of energy required to drive 16 stages of multi-stage flash. This model aims to design and evaluate the amount of thermal energy required for such projects and optimize their performance by calibrating the governing parameters. Furthermore, the 16 stages were simulated via the Ebsilon 13.02 software package to match the results and evaluate the fulfillment of the plant requirements. The temperature drop of the brine stream was 2.34 °C/stage. The top brine temperature was 130 °C. The results show that 29.5 kg/s of superheated steam is required to desalinate 162 kg/s of 2500 kg/s influent mass flow of brine. The effect of water intake temperature was also examined by using Ebsilon. The performance ratio decreased from 5.49 to 2.66 when the water intake temperature decreased from 30 °C to 5 °C.

## 1. Introduction

Many regulations, goals and policies have been issued and organized by different sectors, institutions and governments. Goal No. 6 of the United Nations is one of these policies, which focuses on the delivery of clean and safe drinking water [1,2], given that 300 million people all over the world depend on desalination for potable water [3]. It should be noted here that desalination technology is not limited to the desalting of seawater but can also be used to desalinate brackish water, which contains a lower salt concertation in comparison with seawater (around 15,000 ppm for brackish water). There are two main problems related to large-scale thermal desalination: high fuel consumption and consequent environmental influences, where technologies such as multi-stage flash (MSF) and multi-effect distillation (MED) consume 80–120 kWh of thermal energy and 1.5–4 kWh of electric energy for every cubic meter of desalinated seawater [4]. For example, in the Gulf Cooperation Council (GCC) countries, mainly in Qatar, all of the power generation and desalination capacity is retained by the government (Qatar Electricity and Water Corporation), and fuel is provided to independent water and power production plants at production cost or only marginally higher [5]. Note that the market potential is very high in GCC countries, which have an installed desalination capacity of almost 28 Mm^3^ per day (or roughly 43% of universal capacity) [6]. Many researchers and projects have considered thermal desalination through integration with solar thermal technology (CSP) or hybrid power plants consisting of CSP and auxiliary fossil fuel-driven boilers [7,8]. There is huge variation in the LCOW due to the variation in the capital cost of the chosen technology and the source of energy. In this case, the capital cost will be related to the source of energy, such as a solar field (in the case of solar energy utilization), which can be responsible for providing the required power/thermal energy to the desalination plant, and the cost of the components of the desalination facility itself. According to the literature, many values have been realized in terms of capital cost per cubic meter of daily water production. In [9], the authors reported a value of USD 1700/(m^3^/day), [10] suggested a cost of USD 2200/(m^3^/day), [11] found that the capital cost was around USD 1230/(m^3^/day), and [4] gained a value of USD 2166/(m^3^/day) for MED. The same trend occurred with regard to solar fields and waste incineration power plants, as discussed later. However, it seems to be worth showing some of the variations in the investment cost of the solar field here. For example, in the case of one of the available technologies in CSP, which is the linear Fresnel collector (LFC), it was found that the capital cost ranged from USD 214/m^2^ to USD 235/m^2^ according to [9]. Now, it becomes clear why there is a large variation in the LCOW. In general, according to earlier reports [12,13], the LCOW ranges from USD 2–32/m^3^ for desalination powered by renewable energy [14].

Many researchers and projects have been conducted to investigate the potential of using different kinds of energy sources to supply water desalination facilities with the required energy. A hybrid concept for the source of energy has been applied in some of these investigations in an attempt to moderate the LCOW. The LFC has been used for the direct power or energy supply for thermal desalination plants. When it was connected to MED technology, it was found that the plant production was around 14 × 10^3^ m^3^/day, and the LCOW was 4.2 USD/m^3^. This configuration represents the most expensive option. The LCOW sharply decreased to 2.8 USD/m^3^ when the LFC was hybridized with an auxiliary boiler and MED/TVC was utilized. The production under this arrangement was 5 × 10^3^ m^3^/day. When this system was hybridized with a natural gas boiler, approximately the same cost values were arrived at. The use of parabolic troughs was also investigated. The first and most expensive option was PTC with TES (thermal energy storage) and MSF as the water desalination technology. The LCOW and plant production were 2.6 USD/m^3^ and 2.5 × 10^3^ m^3^/day, respectively. It should be stated here that PTC was also configured with other sources of energy, such as an auxiliary boiler and TES with MED. Another configuration was PTC and TES with MED. These two arrangements have the lowest cost per cubic meter of desalinated water at around 0.9 USD/m^3^.

In general, it can be concluded from all of these studies that PTC technology as a source of the required energy led to the lowest cost for water production in terms of the thermal desalination process.

The main target of the R&D department in this field of work is to find an optimum system for reducing the cost of water production and increase the competitiveness of such a system in the market. In economic terms, economists have detected that doubling the cumulative production volume results in an almost fixed percentage reduction in the unit cost. This means that more energy is required to increase the production of water, and in this case, the mass flow rate of steam (which is the cornerstone of thermal desalination) would be increased.

**Figure 1:**
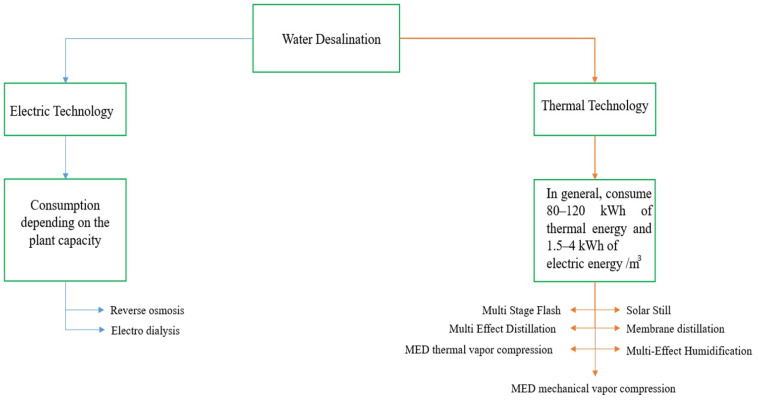
Flow chart diagram illustrating the available water desalination technologies.

Different technologies are available, as can be seen in Figure 1. Thermal desalination technologies have a wide variety of options when it comes to the source of thermal energy (steam) for desalination purposes. Generally, the average thermal energy consumed in such technologies is around 80–120 kwh, and 1.5–4 kwh of electric energy is used per m^3^ of water desalinated [4]. On the other hand, RO technology consumes 2–8.5 kwh/m^3^. This range was specified according to the desalination capacity of the plant. Some literature reported the energy consumption of RO in the range of 0.5–3 kwh/m^3^. This power is required for brackish water, the salinity of which is less than that of seawater [15,16].

The novelty of this work can be summarized in two categories:

The first (design step) was developing a comprehensive model applicable in Excel as a design step for an OT-MSF (once-through multi-stage flash) facility and optimizing its performance by calibrating the governing parameters and variables such as the temperature profiles of the stages and condensers. Furthermore, this model precisely evaluates the amount of thermal energy (steam) required for the brine heater to operate the facility according to the number of designed stages (16 stages in this work), the mass flow rate of the steam, which is the core of energy assessment, and the Non-Equilibrium Allowance (NEA) variable, which is very crucial to assess the vapor temperature inside the stages; consequently, the enthalpy of the vapor can be evaluated, and the pressure profile, which is the driving force of the evaporation process, can be investigated and calibrated.

In the second (simulation step), as an added value, this research increased the efficacy of the design model by applying it (with designed values) to simulation software (Ebsilon) and matching the results. Ebsilon allows converting the design into a virtual desalination plant, predicting the performance and applying optimization. This work allows predicting the economic analysis (which is out of the scope of this research) of such plants in terms of capital and operational costs as an early step before erection in reality.

### 1.1. MSF Configuration

**Figure 2:**
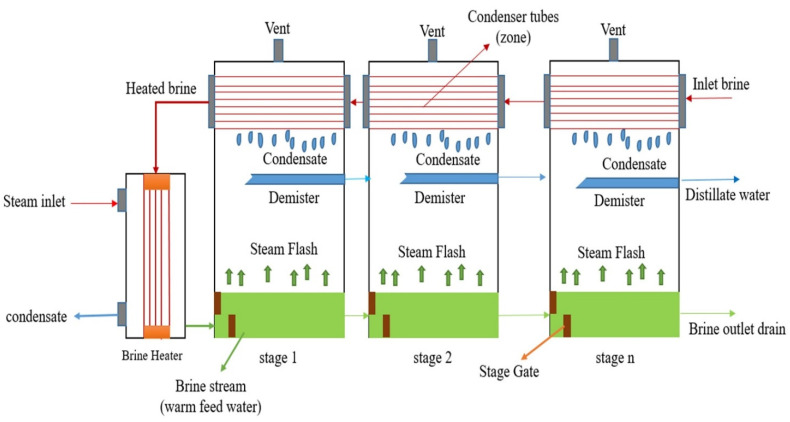
Once-through multi-stage flash.

Two layouts and configurations exist when it comes to designing and creating MSF technology, which are once-through and brine recirculation [17]. Despite the fact that each concept has its own advantages, the once-through (OT) concept is easier to model and simulate. For this reason, the OT technique was chosen in this research to be connected to a power plant, as shown in Figure 2. In the MSF procedure, the separation is accomplished by evaporating some of the feed (brine or seawater) in each stage by flashing. The hot feed seawater flowing into each stage encounters a lower pressure than its own vapor pressure; it then flashes off, producing vapor on one side and cooling the brine flow on the other side [18].

The flashing process is unbroken, stage after stage, due to the continuous reduction in stage pressure afforded by the descent in the pressure of the brine flowing through the brine orifices. The advantages of using multi-stage flash distillation for desalination include the quality of the water produced, which contains less than 10 mg/L TDS. The salinity of the feed water does not have much impact on the process or costs of MSF. It can be united with other processes, e.g., by using the heat energy from an electricity generation plant, as is described in this work, by coupling MSF technology with a hybrid power plant.

Many differences exist between the two configurations, whereby the OT design is not only easier in concept and design but also has a higher performance ratio, especially in the case of large-scale projects. According to [19], three major features verify the advancement of the OT design—the performance ratio, the top brine temperature and the brine loading, all of which are superior. For example, the performance ratio is just like efficiency, and it can reach 12 for the once-through design. The top brine temperature can increase to 130 °C, which means efficient absorption of thermal energy (heat) from heating steam. It should be noted here that the main disadvantage of the OT design is its high consumption of chemicals for the pretreatment of water. On the other hand, the elimination of the heat rejection section and the recirculation pump, with all of the associated controls and civil work, means lower capital investment and simpler operation than is the case with the brine recirculation (BR) configuration. In any case, the selection of a specific configuration depends mainly on economic and operational considerations.

The BR practice uses very few chemicals for makeup pretreatment. The consumption of chemicals depends on the volume of seawater to be treated, as well as the concentration ratio. While dosing rates, in ppm, are lower for the OT process, as the coolant seawater has the equivalent feed concentration, the large intake flow rate calls for a higher rate of chemical dosing than in the case of BR. The net result is higher overall chemical consumption in the OT process. In the BR process, deaeration of the pretreatment is accomplished in a deaerator, which is integrated with the last rejection stage. Further, deaeration takes place in the first few stages of the OT process. This is a disadvantage since the amounts of noncondensable gases released, including oxygen, are much higher than what is released by recycling in the BR process [20].

### 1.2. Process Description

In this part, the process by which seawater goes through the desalination stages is described. As mentioned above, the first level of the process is the start-up of the water supply to the station. Of course, the source of seawater in the case of Jordan is the Red Sea. The temperature of the intake was around 25–30 °C; therefore, a preheater was added to the simulated plant to moderate the intake seawater temperature through the year, whereby the influent seawater into the plant was fixed at approximately 45 °C. Some chemicals are added to the feed water (anti-scaling, chlorine, and anti-foaming). Chlorine is added to the seawater feed at the source to avoid bio-fouling inside the condenser tubes [21]. The water is then pumped to the deaerator and then into the condenser tubes (the heat recovery section) to increase its temperature, stage by stage. After warming the seawater feed in the condenser tubes, part of this feed water is evacuated to the sea (known as cooling water), while the rest of the feed water is delivered to the deaerator (known as makeup water) to complete the distillation process.

The feed seawater enters the last stage (stage 16 in this work), crossing the condenser tube bundles stage by stage until reaching the first stage; then, it passes through the brine heater to harvest its maximum energy from the steam, and the feed water leaves the brine heater to reenter the stages (at the first stage) but on the lower part this time (brine stream flow). It should be noted that its temperature is highly increased by passing through the brine heater. There is a temperature difference between the feed water stream flow in the condenser tubes (upper section) and brine stream flow (lower section), and the noncondensable gas vent, which is accompanied by a pressure drop in each stage, makes the pressure of the brine flow reach its saturation level (saturation pressure), which pushes the upper layer of the brine stream to be evaporated. The feed water temperature in the condenser section is lower than that of the brine stream flow, and due to that, the formed vapor will condense at the outer surface of the condenser tube bundles and be collected in the demister cumulatively, stage by stage. The feed water temperature increases evenly to the temperature drop of the brine stream, which, in this research, was 2.34 °C in each stage at a constant rate, as explained later.

#### 1.2.1. Deaerator

The makeup water stream is pumped into a deaerator turret to spoil bicarbonates for the separation of dissolved gases such as oxygen, nitrogen and carbon dioxide. Such gases have a negative influence on the heat transfer process due to their low thermal conductivity. Furthermore, chemical corrosion in numerous locations of the plant can be caused by carbon dioxide and oxygen.

#### 1.2.2. Condenser Tube Section and Brine Heater

After the deaerators, the treated feed water (makeup) is pumped to the heat recovery section (condenser tubes). The brine then arrives at a brine heater, where it is heated to its maximum temperature using steam from a boiler. The simulated facility was able to produce around 13,850 m^3^/day of distillate water in a process consisting of 16 stages (evaporators). The results indicate that the temperature of the top brine (T_0_) reached its maximum value during the first stage (around 95 °C) and then started to decrease in each stage at a rate of 2 °C per stage. It ultimately dropped to 55 °C in stage 16. As can be seen here, the temperature in the effluent stage of the plant was high. Therefore, as mentioned before, a preheater was added to moderate the intake water temperature and to harvest the heat in the brine before returning it to the water source.

In terms of the mass flow rate, desalinated water was accumulated stage by stage. At stage 1, the quantity of condensed water in the tray was around 3 kg/s. At stage 2, it became 6.3 kg/s, and by stage 16, it was around 97 kg/s. At the same time, the mass flow rate of the brine water (seawater) was declining due to the evaporation process in each stage. After the evaporation in stage 1, the mass flow rate declined to 1497 kg/s and continued to decline until stage 16 (the driven flow rate of saltwater is 1500 kg/s), where it was almost 1400 kg/s. It can be concluded that the mass equilibrium of the evaporated and condensed water was around 3 kg/s in each stage.

It should be distinguished here that the salt concentration of the seawater increased from 42,000 mg/L to 45,000 mg/L, because during the water evaporation through the stages, the water shifts from a liquid phase to a saturated water vapor phase, leaving behind an increased concentration of salt in the brine water in the lower stages of the MSF process. It should be noted here that the water formed is ready to use in the industrial sector. If the water is planned to be used as a source of potable water, post-treatment is required in order to comply with local health guidelines, preventing the risk of biological growth. A number of drinking water regulations and guidelines define the concentration limits for several substances that are potentially hazardous to human health. In terms of brine discharge, it will of course contain a high concentration of salt and chemical compounds such as calcium bicarbonate due to the pretreatment process of the water. In addition, working with high temperatures in the evaporation stages means that the brine must be well-treated before being recirculated to the seawater source.

Note that the desalinated water flows from one tray to the next one. Finally, the distillate is driven to storage tanks, where it is chemically treated by chlorination and adjusted in terms of its pH value.

#### 1.2.3. Vacuum System

It is vital to use a vacuum system to remove noncondensible gases from the plant and to maintain the required reduced pressures. The usual system is a two-stage steam jet ejector system with a barometric vent condenser, an inter-condenser and a final condenser.

## 2. Methodology and Design

The mathematical model was adopted from [22] to include the complete material balance, stage and condenser temperature profiles, stage material and salt balance, condenser and brine heater heat transfer areas, stage dimensions, and performance factors.

### 2.1. Overall Material Balance

The whole system is treated as a block. In terms of mass balance, there is one input and two yields to the feed seawater, M_f_, and two output streams, the desalinated product, M_d_*,* and the rejected brine, M_b_. Therefore, the overall material balance equation is:

(1) $$M_{f}={\;M}_{d}+{\;M}_{b}$$

The total salt balance is given by:

(2) $$X_{f}M_{f}={\;X}_{b}M_{b}$$

where X is the salt concentration (ppm).

### 2.2. Stage and Condenser Temperature Profiles

Four temperatures are the governing parameters in the OT-MSF process. These are the temperatures of the steam, T_s_, the temperature of the brine leaving the preheater (top brine temperature), T_0_, the temperature of the brine leaving the last stage, T_n_, and the temperature of the feed seawater, T_f_. The temperature drop per stage, ΔT, is obtained from the relationship. Note that a linear outline for the temperature is assumed for the flashing brine and the seawater flowing inside the condenser tubes:

(3) $$\Delta T=\left(T_{0}-{\;T}_{n}\right)/n$$

where n = number of stages.

A general expression is developed for the temperature of stage i:

(4) $$T_{i}={\;T}_{0}-i\;\Delta T$$

A common equation is used for the condenser temperature in stage i:

(5) $$t_{i}={\;T}_{f}+\left(n-\left(i-1\right)\right)\Delta t$$

The main driver of vaporization inside each stage is the pressure drop of the brine, which is a function of the temperature. It should be noted that the temperature of the feed seawater, M_f_, which runs inside the condenser tubes, rises by ∆t in the condenser of each stage. This temperature increase, ∆t, is equal to the decrease in the brine temperature in each stage, ∆T.

### 2.3. Flash Vaporization and Latent Heat

After increasing the temperature of the brine to the design temperature point in the brine heater, it enters the first stage series of the MSF stages, and the flash vaporization process starts due to the difference in temperature between the brine and the flowing feed water in the condenser tubes. The latent heat spent by the flashing vapor is set to be equivalent to the decrease in the brine sensible heat:

(6) $$D_{1}={\mathrm{yM}}_{f}$$

where D_1_ is the quantity of flashing vapor shaped in the first stage, M_f_ is the feed seawater flow rate, and y is the specific ratio of sensible heat to latent heat and is equal to:

(7) $$y={\;C}_{p}\;\Delta T/\lambda_{\mathrm{av}}$$

where Cp is the specific heat capacity (KJ/Kg.K), and λ_av_ is the average latent heat (KJ/kg), in other words, the enthalpy of vaporization, which is the amount of energy required to vaporize a unit mass of saturated liquid at a given temperature or pressure. It decreases as the temperature or pressure increases and becomes zero at the critical point. For the desalination plant, it can be calculated through:

(8) $$T_{\mathrm{av}}=\left(T_{0}+T_{n}\right)/2$$

Note that by increasing the pressure designed for each stage of the desalination plant, the mass flow rate of the desalinated water increases due to the decrement in the latent heat required to vaporize the same amount of brine.

According to [22], the top brine temperature, T_0,_ and the brine leaving the last stage, T_n_, were taken as 93 °C and 54 °C, respectively. T_av_ becomes 74 °C (assumed to be 75 °C) by applying thermodynamic property tables to determine the pressure and vaporization enthalpy. The phase nature of the brine is saturated liquid. Table 1 presents the design parameters used in the desalination plant.

As the maximum brine temperature and the brine temperature in the last stage are known, the temperature drop per stage is also known. The temperature drop per stage was found to be equal to 2.3 °C at a constant rate for 16 stages.

(9) $$D_{i}={\;M}_{f}y\;{\left(1-y\right)}^{\left(i-1\right)}$$

This equation can be used to calculate the amount of distillate water in any stage. The total amount is calculated by taking the summation of D for all stages.

The targeted amount of desalinated water can also be designed and calculated as the accumulated mass flow stage by stage as follows:

(10) $$M_{d}={\;M}_{f}\;\left(1-{\left(1-y\right)}^{n}\right)$$

The flow rate of the brine stream exiting stage i is given by:

(11) $$B_{i}={\;M}_{f}-D_{i}$$

The salt concentration in the brine stream leaving stage i is given by:

(12) $$X_{i}={\;M}_{f}X_{f}/B_{i}$$

The flow rate of the heated steam required to flow in the preheater (the brine heater) can be calculated as follows:

(13) $$M_{s}=M_{f}C_{p}\;\left(T_{0}-t_{1}\right)/\lambda_{s}$$

The temperature and pressure of the steam, which is the main source of energy required to drive the desalination facility, have already been specified as 130 °C and 2 bar, respectively, in Ebsilon, according to the data available in the literature and practical experience from existing projects. In this case, the temperature and pressure of the steam are specified, and its phase (as superheated steam) is also available. Turning back to the property tables, the enthalpy (energy content) can be specified at around 2720 KJ/kg. According to the iterative solution provided by Ebsilon, the mass flow rate was found to be equal to 29.2 kg/s, whereas when using Equation (13) above, it was 25 kg/s. The difference in findings is due to a gap in the required energy in the preheater (the brine heater). In other words, in terms of the energy conservation law, the amount of heat extracted from the steam to increase the temperature of the brine (in the condenser zone) after passing through all stages and reaching the brine heater means that its temperature reaches 86.5 °C with 2500 kg/s mass flow. The energy held in this stream of water was around 865.942 MW, with the enthalpy around 356 KJ/kg. The designed maximum brine temperature (as mentioned before, this is the temperature of the brine after passing through the brine heater) was 93 °C. Once again, with 2500 kg/s, the brine will hold 932.022 MW, where enthalpy is around 373 KJ/kg. The increment in energy is transferred to it as it passes through the brine heater. The amount of heat extracted from the steam in the brine heater is as follows:

(14) $${\underset{m}{˙}}_{0}h_{0}\;-\;{\underset{m}{˙}}_{1}h_{1}=\mathrm{extracted}\;\mathrm{heat}\;(\mathrm{energy})$$

**Figure 3:**
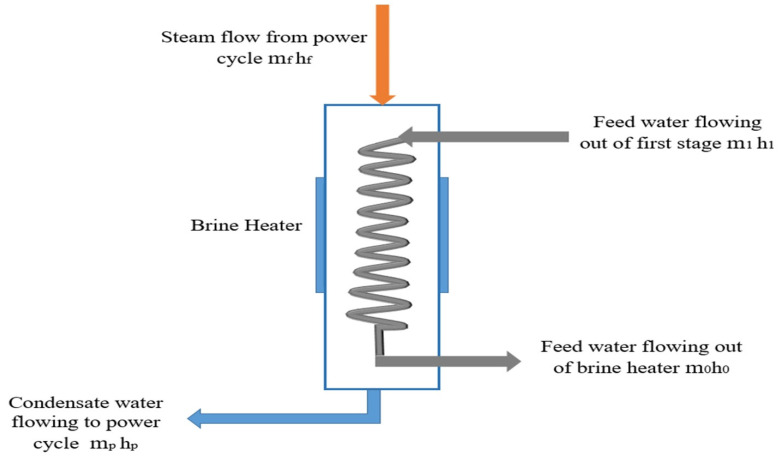
Brine heater: m_1_ h_1_, mass flow and enthalpy of feed water; m_0_h_0_, mass flow and enthalpy of feed water coming out of brine heater; m_p_ h_p_, mass flow and enthalpy of condensate water; and m_f_ h_f_, mass flow and enthalpy of steam.

As can be seen in Figure 3, the amount of heat extracted from the steam is as illustrated in Equations (14) and (15). As was calculated with 25 kg/s as the steam flow (m_f_), this stream will hold 68,225 kW (at 130 °C and 2 bar, it has 2729 KJ/kg), and the condensate stream line holds 11,675 KW (at 25 kg/s, 111 °C and 1.5 bar). The amount of energy released in this case is 56,550 KW:

(15) $${\underset{m}{˙}}_{f}h_{f}\;-\;{\underset{m}{˙}}_{p}h_{p}=\;\mathrm{energy}\;\mathrm{released}$$

This does not cover the amount of heat required for the brine stream, where the required heat to raise the temperature of the brine from 86.5 to 93 (at 2500 kg/s) is 66,080 KW. Consequently, the mass flow of steam was increased to 29 kg/s to cover the required energy.

### 2.4. Brine Heater (Preheater) and Condenser Heat Transfer Areas

The brine heater area can be calculated as follows:

(16) $$A_{b}={\;M}_{s}{\;\lambda }_{s}/(U_{b}\;\left(\mathrm{LMTD}{)}_{b}\right)$$

The logarithmic mean temperature difference has to be found:

(17) $${\mathrm{LMTD}}_{b}=\frac{\left((T_{s}-{\;T}_{0}\right)-\left(T_{s}-t_{1}\right))}{\ln\left[\frac{T_{s}-T_{0}}{T_{s}-t_{1}}\right]}$$

The overall heat transfer coefficient can also be found as follows:

(18) $$U_{b}=1.7194+3.2063*\;10^{-3}T_{s}+1.5971*10^{-5}T_{s}^{2}-1.9918*\;10^{-7}{\;T}_{s}^{3}$$

The next step is to determine the heat transfer area of the condenser part. It is assumed that the area in each stage is equal. Consequently, the heat transfer area for the condenser for any stage can be taken to be the same as in any other stage.

(19) $$A_{c}={\;M}_{f}C_{p}\;\left(t_{1}-t_{2}\right)/\left(U_{c}{\;\mathrm{LMTD}}_{c}\right)$$

Again, Equation (19) above needs LMTD and U values for the condenser. These can be evaluated as follows:

(20) $$U_{c}=1.7194+3.2063*\;10^{-3}{\;T}_{V_{1}}+1.5971*\;10^{-5}T_{V_{1}}^{2}-1.9918*10^{-7}T_{V_{1}}^{3}$$

(21) $${\left(\mathrm{LMTD}\right)}_{c}=\frac{\left(T_{V_{1}}-t_{1}\right)-\left(T_{V_{1}}-t_{2}\right)}{\mathrm{Ln}\left[\frac{\left(T_{V_{1}}-{\;t}_{1}\right)}{\left(T_{V_{1}}-t_{2}\right)}\right]}$$

A new term appears in the form of $T_{V_{1}}$. This is the condensing vapor temperature (at which water vapor will be condensed), and it can be found as follows:

(22) $$T_{V_{1}}={\;T}_{1}-{\;\mathrm{BPE}}_{1}-{\mathrm{NEA}}_{1}-\Delta T_{d_{1}}$$

$\Delta T_{d_{1}}$ is the temperature drop in the demister, which is assumed to be zero. ${\mathrm{BPE}}_{1}$ is the boiling point elevation:

(23) $${\mathrm{BPE}}_{1}={\;X}_{1}\;\left(B+\left(X_{1}\right)\;\left(C\right)\right)10^{-3}$$

The coefficients B and C in the correlation for the boiling point elevation are:

(24) $$B=\left(\left(6.71+6.34*\;10^{-2}T_{1}\right)+9.74*\;10^{-5}T_{1}^{2}\right)*\;10^{-3}$$

(25) $$C=\left(22.238+9.59*10^{-3}T_{1}+9.42*10^{-5}T_{1}^{2}\right)*10^{-8}$$

Equation (22) contains another important term that needs to be evaluated: NEA (Non-Equilibrium Allowance). This parameter considers the discrepancy between the real and ideal evaporation (brine flashing). This difference is caused by three different factors: First is the irreversibility of the real process. The flashing brine keeps a portion of the overall energetic content of the inlet brine that is therefore not available for steam production. Second, the transformation (the phase change) occurs in a finite time and is therefore not at equilibrium; the third is overpressure in the lower layer of brine flowing through the flashing pool due to the onset of a hydraulic head. As a result, thermodynamic equilibrium conditions are not reached, and the temperature of the brine will be higher than the ideal one.

The term NEA needs to be inserted into Equation (22) to find the temperature of steam in the stage. This is a function of 6 parameters:

(26) $$\mathrm{NEA}=f\left(T_{\mathrm{in}},{\;T}_{\mathrm{out}},\;L,\;W,{\;m}_{\mathrm{in}},\;H\right)$$

According to [22], it can be found by the following equation:

(27) $${\mathrm{NEA}}_{1}={\left(0.9784\right)}^{T_{0}}{\left(15.7378\right)}^{H_{1}}{\left(1.3777\right)}^{V_{b}*10^{-6}}$$

Equation (27) requires V_b_ and H_1_, which denote the brine mass flow rate per stage width and the brine height, respectively. This allows us to determine the length and width of the stage, the vapor density and the vapor velocity in the demister. These were experimentally evaluated in [22]. The temperature of the steam formed in a stage can be evaluated as follows:

(28) $$m_{\mathrm{Bi}}h_{\mathrm{Bi}}-m_{\mathrm{Bo}}h_{\mathrm{Bo}}={\;m}_{\mathrm{st}}h_{\mathrm{st}}$$

Note that m_st_ can already be calculated by Equation (9), and $m_{\mathrm{Bi}}h_{\mathrm{Bi}}-m_{\mathrm{Bo}}h_{\mathrm{Bo}}$ is obtainable, where the mass flow of brine $m_{\mathrm{Bi}}$ is assumed as a design requirement, and the temperature of the brine can also be found (for the first stage, this is the maximum brine temperature, T_Bi_), and $m_{\mathrm{Bo}}h_{\mathrm{Bo}}$ is found after finding ∆T per stage. It is then possible to directly determine the precise value of enthalpy of the steam inside the stage by returning to the thermodynamic property table. By iteration, the temperature of the steam can be found by:

(29) $$H_{v}=h_{\mathrm{st}}=2500.15+1.947036\;T-0.00195{\;T}^{2}$$

However, to continue with the steps of the model to determine the condenser area, there is no need to evaluate the NEA where the steam temperature was found. It should be noted here that NEA was evaluated by adapting two methodologies. By evaluating the value of the steam temperature in the first stage and inserting it into Equations (20) and (21), the area of the condenser can be calculated.

The total heat transfer area can be calculated using:

(30) $$A={\;A}_{b}+{n\;A}_{c}$$

where n is the number of stages. The performance ratio of the whole plant is given by:

(31) $$\mathrm{PR}=\frac{M_{d}}{M_{s}}$$

**Figure 4:**
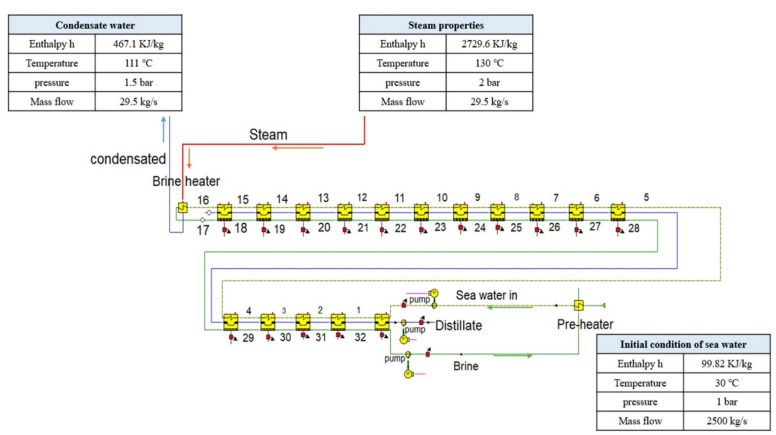
Once-through MSF desalination plant consisting of 16 stages simulated in Ebsilon.

Figure 4 shows the desalination plant simulated in Ebsilon. The illustrated figure is related to a standard case in which the temperature of the seawater is taken as 30 °C. As can be seen, three main streams are illustrated: First is the steam flow, which comes from the power block of the power plant (the second steam turbine) to inject its thermal energy into the feed seawater through the brine heater. The steam line conditions are 130 °C, 29.5 kg/s and 2729 KJ/kg in terms of temperature, mass flow rate and enthalpy, respectively. Second, in terms of condensate, this stream flow represents the compensated water flow returning to the power cycle as liquid water, which has a temperature and mass flow of 111 °C and 29.5 kg/s, respectively. Third, the seawater stream flow before entering the preheater is at 30 °C, 1 bar, 99.8 KJ/kg and 2500 kg/s in terms of temperature, pressure, enthalpy and mass flow, respectively.

## 3. Results and Discussion

**Figure 5:**
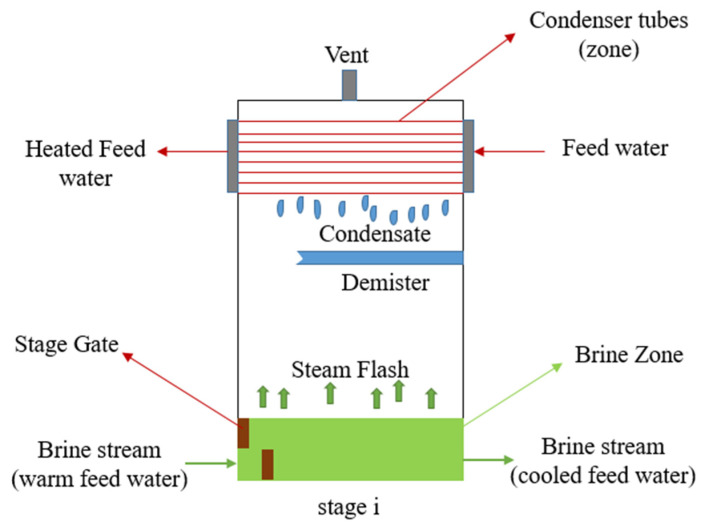
MSF desalination stage.

It can be said that there are two major spots (fields) in each stage of the desalination plant: the first is where the heat (thermal energy) is generated, driven by the law of energy conservation between the inlet and the outlet of the brine stream; the second is the condenser tubes in the upper part of the stage. The feed water flows from the last stage (stage 16 in this research), where the intake water (from the seawater source) enters the plant to the first stage, which lies just before the brine heater. The brine heater, which is the core of the facility, works to increase the temperature of the feed water to the designed top brine temperature after receiving it from the condenser zone from the first stage. The temperature of the feed water (seawater) is increased in the brine heater and delivered to the first stage again at a higher temperature (known as the top brine temperature) and, of course, with higher energy (as mentioned before, the top brine temperature for this research was taken to be 93 °C). In each stage, two thermodynamic processes take place simultaneously: First, energy is generated in the lower part of the stage from the brine stream (heated feed water) due to the difference in energy in and out of the stage and the pressure drop of the brine stream per stage. The amount of heat (thermal energy) released is known as latent heat of vaporization *(*$h_{\mathrm{fg}}\;\mathrm{as}\;\mathrm{it}\;\mathrm{is}\;\mathrm{referred}\;\mathrm{to}\;\mathrm{in}\;\mathrm{thermodynamic}\;\mathrm{books})$, which makes a small amount of the brine water (feed water) start to vaporize and convert into steam. Second, the generated steam is condensed and converted into salt-free water in the demister, and heat is released due to condensation and phase change (from steam to distilled water) and is absorbed by the feed water stream flowing in the condenser tubes in the higher portion of the stage. Thus, the feed water in the condenser zone also increases. Figure 5 explains the condenser and brine zones to illustrate the heat transfer process inside the desalination stage.

The assumption of the temperature change that was considered earlier makes the temperature profile in both the condenser zone and brine zone systematic and linear.

**Figure 6:**
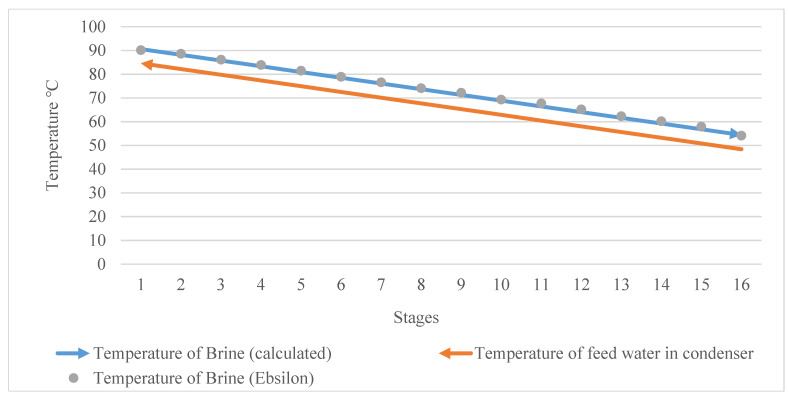
Temperature distributions of feed water in condenser and brine zones.

The brine temperature zone has two lines (the dotted line and the blue line in Figure 6). The dotted line presents the results of the brine zone temperature for 16 stages from the simulation (Ebsilon), while the blue line shows the calculated temperature profile by using the equation model. It can be clearly seen that the designed temperature profile (calculated) perfectly matches the results of the simulation. This matching is related to the tracking of the designed temperature drop, ∆$T_{i}$, per stage related to the brine zone (this was calculated to be 2.34 °C), which was assumed to be equal to the temperature increment of the feed water in the condenser zone (condenser tubes) (∆$t_{i})$. It can be stated that there is no deviation between the designed temperature and the temperature from the simulation. The gap between the design and the simulation (the deviation) can be seen in the profile of the pressure drop. This is explained later.

Regarding the brine zone, the temperature of the brine stream (the feed water) dropped from 93 °C (the influent temperature in the first stage) to around 90.5 °C. The temperature drop was around 2.34 °C. In other words, the designed temperature drop, ∆$T_{i}$, can be considered the temperature step drop for the brine stream zone and the temperature step increment for the condenser zone (condenser tubes). Consequently, the temperature of the brine stream dropped from 93 °C (top brine temperature) to around 54.5 °C in the last stage. On the other hand, the feed water entered the desalination plant at the last stage (stage 16) at 48.5 °C, not at 25 °C or 30 °C (this point is explained later), and reached around 86 °C in the first stage before entering the brine heater.

### 3.1. Flash Vaporization and Latent Heat

As explained earlier, the MSF desalination facility involves very tricky technology. The energy is harvested twice, and the latent heat of vaporization is ensured by energy conservation in the brine zone. This is due to the temperature difference inside the stages between the condenser zone and the brine zone. The flashing steam starts to condense, and the energy released by condensation is harvested by the feed water in the condenser zone.

**Figure 7:**
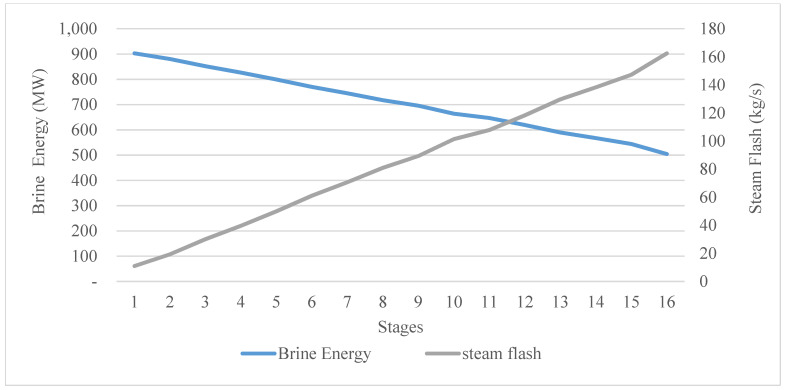
Brine energy and steam flash per stage.

**Figure 8:**
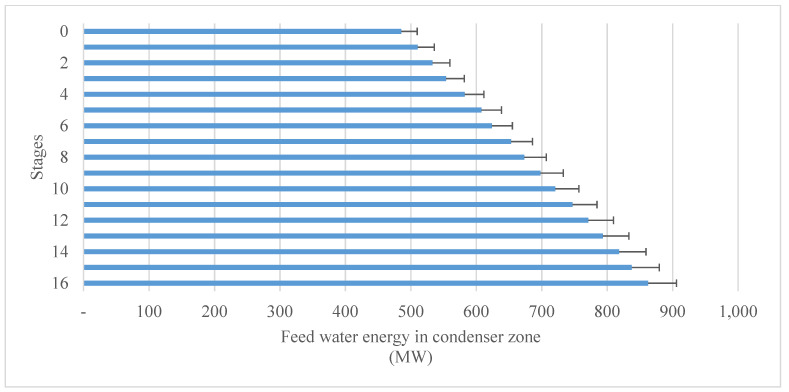
Energy increment of feed water in condenser zone; stage (0) denotes the initial condition (flow of feed water before entering stage 16 of the plant as intake water).

Before entering the first stage, the brine water (feed water) holds a huge amount of energy. It starts to exploit this energy in stage 16 in the condenser zone before reaching stage 1 (the condenser zone). It then enters the brine heater to harvest more energy and exits the brine heater at the designed top brine temperature. Before entering the first stage of the brine stream (feed water), it has its maximum energy, as can be seen in Figure 7. Before entering the first stage, it contained around 932 MW of energy. In fact, this huge amount of energy can be attributed to the mass flow rate of the feed water, which needs to be desalinated in the whole plant. The designed mass flow rate of the feed water (2500 kg/s) entered the plant at 48.5 °C and had 194 KJ/kg. Thus, according to these initial conditions, before entering the plant, the feed water held around 485 MW of energy, as can be seen in Figure 8.

The energy of the feed water increased, stage by stage, from 485 MW to around 862 MW at the effluent from stage 1 and just before entering the brine heater. The temperature of the feed water increased from 48.5°C to 86.5 °C before the brine heater. It can be concluded that around 377 MW of thermal energy was harvested by 2500 kg/s of the feed water (seawater) stream flowing in a series of condenser zones (condenser tubes) in 16 stages of the water desalination plant.

**Figure 9:**
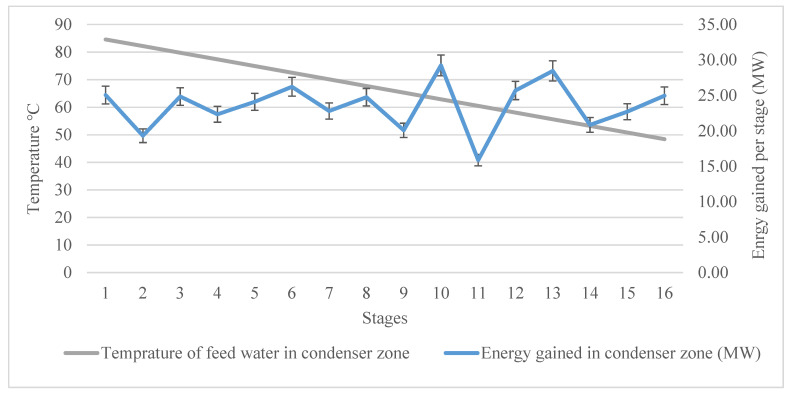
Thermal energy harvested in the condenser zone and the temperature increment of the feed water.

The distribution of energy gained in each stage of the plant is illustrated in Figure 9. The energy gain was quite different from stage to stage. It can be seen that it was not linear or exponential accumulative. This can be attributed to the unsystematic pressure drop, as mentioned before, when tracking the temperature profile of the temperature drop in the design steps. Starting from stage 16, where the feed water flows into the desalination plant, the feed water exploited around 25 MW of thermal energy, while in stage 14, it was around 20.8 MW. The minimum amount of thermal energy harvested was in stage 11 at around 15 MW, while the maximum was in stage 10 at around 29 MW.

### 3.2. Desalinated Water and Brine Flow Rates

The mass flow rate of brine water in the lower part of the stages (the brine zone) is reduced stage by stage due to the evaporation process inside the stages. The evaporation draws a small fraction of the mass flow rate of the brine water (the feed water) to produce steam, followed by distillate water. The amount of distillate water constituted in the demister is equal to the amount of steam formed (brine water evaporated).

**Figure 10:**
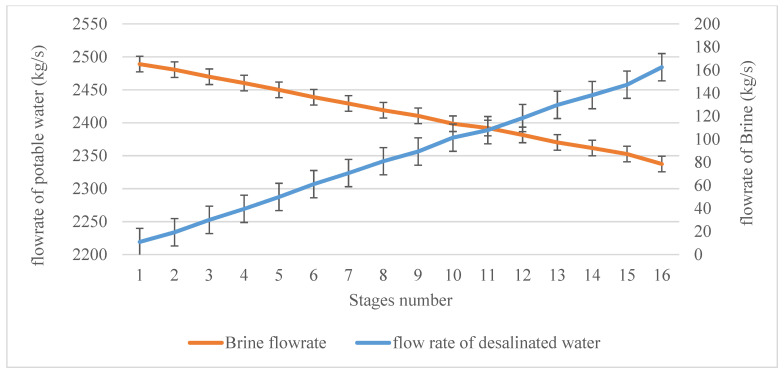
Mass flow rates of brine water (feed water) and desalinated water in the demisters.

The mass flow of brine water (feed water) in the first stage was 2500 kg/s. This exited from the first stage at 2489 kg/s, with around 11 kg/s being evaporated and converted into distillated water in the demister. After passing through the second and third stages, the mass flow of the brine was 2480.6 kg/s and 2469.8 Kg/s respectively, as shown in Figure 10. It can be concluded that the mass flow must decrease after passing through each stage, but not at a constant rate. This is also due to the effect of the pressure drop from stage to stage. At the last stage of the plant (stage 16), the mass flow of brine water was 2337.5 kg/s, and the net reduction in the mass flow of brine water was around 162.5 kg/s. On the other hand, evaporated steam started with 11 kg/s at the first stage and ended with around 13 kg/s at stage 16. It should be noted that the accumulated distillate water from all 16 stages was 162.5 kg/s, thus exactly verifying the law of mass conservation in any system. This also provides an accurate check with regard to the amount of water that can be formed from a desalination plant in the earlier steps during the design phase. This can be determined by tracking and following up the mass flow reduction of brine water in the plant to predict the possible amount of water that can be desalinated. If the amount of desalinated water is less or more than the amount of reduced mass flow of brine water, in this case, there will be a mass loss in the plant, and this is not interpretable, especially in the design phase.

**Figure 11:**
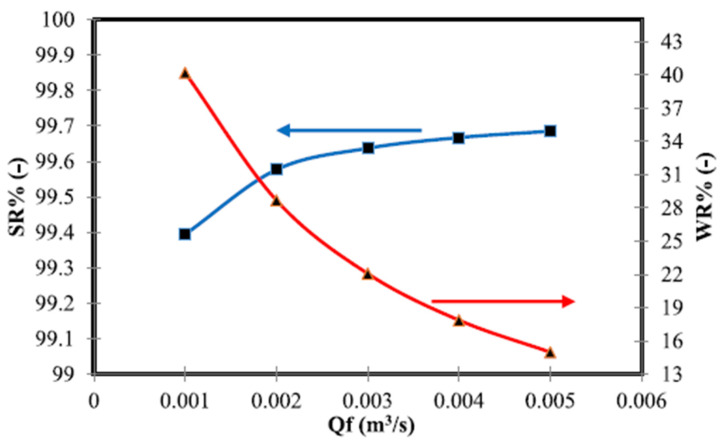
Effect of feed water flow rate on the water distillate and salt concentrations (WR; water recovery, SR; solute rejection, $Q_{f}$; feed water flow rate), adapted from [23].

To illustrate the strengths of MSF in terms of desalinated water generation, it can be compared with the performance of RO and MED, which are the main technologies governing the desalination domain. Al Adwani et al. [23] studied the sensitivity and the effect of different operating parameters on the performance of RO desalination plants. The effect of seawater flow rate was one of the examined parameters that are directly related to the results of this research in terms of distillate water and brine flow rates. They found that increasing the mass flow rate of feed water (water intake) caused a slight increase in brine, which makes sense, while increasing the flow rate of feed water was accompanied by a significant decrease in water recovery (desalinated water). They stated that when increasing the mass flow of feed water from 0.001 m^3^/s to 0.005 m^3^/s, water recovery decreased by around 62%. As can be seen in Figure 11, increasing the feed flow rate simply means a lower residence time of the fluid inside the membrane module, which would significantly reduce the filtration time and cause a reduction in water flux.

It is worth noting here that many researchers compared the main desalination technologies (MSF, MED and RO) in terms of unit size and distillate quality. Using data from [2,24,25], a comparison was made to present the key differences between the main technologies, as can be seen in Table 2.

**Figure 12:**
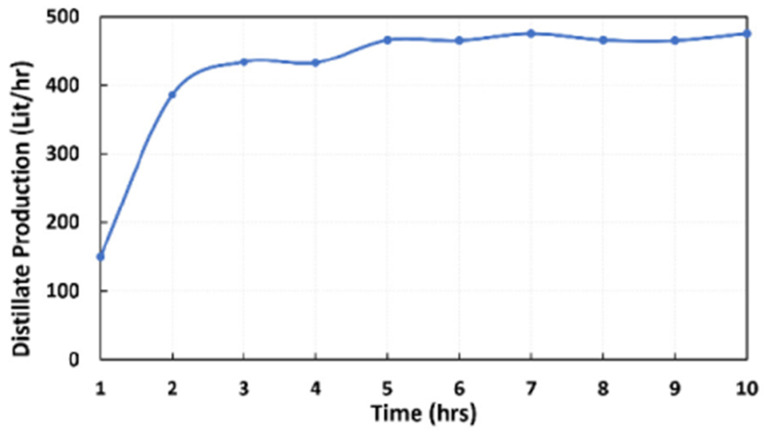
Distillate water production for one cell of a MED facility during 10 operation hours, adapted from [26].

As can be seen, MSF has the advantage of desalinating more water with a very low concentration of salt. It should be noted here that MED is particularly affected by scaling on pipes in comparison with MSF; due to that, several research projects about MED have been realized, investigating lower temperatures to reduce scaling issues and the corrosion of the pipes, such as MED-TVC (Multi-Effect Distillation–Thermal Vapor Compression), MED–BD (Multi-Effect Distillation–Back Side Demister) and many others that are out of scope of this research [2]. This scaling problem explains the limited MED stages. Aly Sh. et al. [26] presented a new design for the tube bundle of a MED facility consisting of three cells (stages) simulated and installed as an advanced pilot project in Qatar with a nominal capacity of 25 m^3^/day (0.28 kg/s)–42 m^3^/day (0.48 kg/s) per cell at a top brine temperature of 65 °C. The results show that distillate production varies can reach 475 L per hour as its maximum after 5–6 h to reach steady state, as shown in Figure 12, where the desalination rate in the first couple of hours was 100 L/hr. This emphasizes the limitation of MED technology for water desalination. Note that they assumed that the top brine temperature was constant, while it normally decreases, leading to a consequent decrease in the distillate water. Around 11.4 m^3^/day of distillate water can be determined from the mentioned results by assuming that steady-state conditions were reached after the first hour, while the simulation results of this research paper show that it is possible to produce around 13,850 m^3^/day of distillate water, which can match the high consumption rate of water.

### 3.3. Salt Concentration of Brine Water

One of the drawbacks of the water desalination concept, regardless of the technology used (RO, MED or MSF), is that the stream of water exposed to desalination will contain more salt after passing through the whole desalination plant. As mentioned before, many researchers have attempted to deal with and manage the effluent brine water and have recognized the importance of higher salt concentration in order to reduce its negative environmental and ecological impacts. Nowadays, this concept is considered a benchmark for many research topics worldwide.

**Figure 13:**
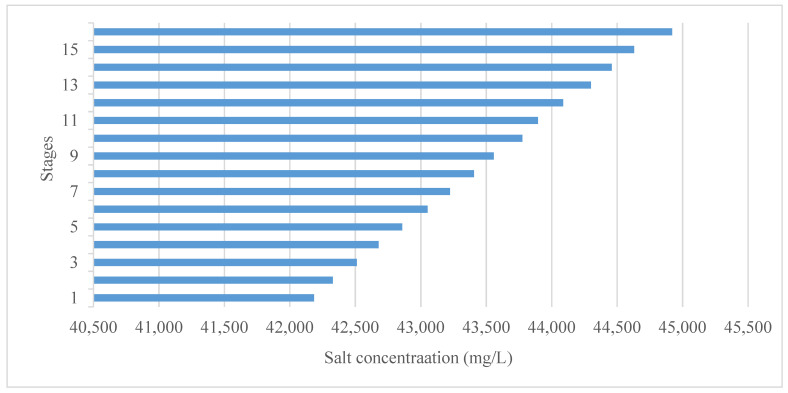
Salt concentration change in brine water (feed water).

As an initial condition, the feed water is pumped from the Red Sea, and the salt concentration is assumed to be 42,000 mg/L. This salt concentration already exists in seawater and represents the concentration prior to entering the first stage of the plant. After passing through the first stage, the salt concentration increased by 185 mg/L to become 42,185 mg/L and then became 42,328 mg/L and 42,512 mg/L after passing through the second and third stages, respectively. At the last stage, the salt concentration was around 44,920 mg/L, as can be seen in Figure 13. The total increase with regard to the concentration of salt was around 2920 mg/L from the first to the last stage. It can be concluded that there was an increase in salt in each liter of brine water of around 182.5 mg/L per stage. These results show the potential to determine the right concept or methodology to deal with the salt increase as a result of the desalination process.

Innovative efforts have been introduced for ways to treat or use brine in order to minimize or eliminate the negative environmental impacts associated with brine disposal [27] and/or to partially or fully balance the economic costs associated with brine disposal [28]. The exploited methods that have been developed to moderate the salinity and chemical compounds accompanied with brine can be summarized as follows:

1. The involvement of brine with other water sources of lower salinity (e.g., treated wastewater or power-plant cooling water) can reduce brine salinity by dilution [29].
2. Pressurized dispersion nozzles can support the mixing of brine waters with receiving waters, restricting bottom ponding.
3. Techniques such as bipolar membrane electrodialysis (BMED) can convert brine into acid and base yields for reprocessing, such as NaOH and HCl [30].

Other potential economic opportunities related to brine production have also ignited a wave of innovation in brine management in an attempt to turn an environmental problem into an economic prospect. Sequential biological concentration (SBC) is one these techniques and has been used to create economic benefits by using saline drainage and by converting it to manageable drainage. Integrating agriculture and aquaculture systems is another proposed methodology with regard to using the SBC as salty drainage water sequentially. This has potential for commercial, social and environmental achievements. Reject brine has also been used for aquaculture, with a 300% increase in fish biomass being achieved. Reject brine has also been successfully used for Spirulina cultivation and for the irrigation of halophytic forage shrubs and crops, even though this technique is powerless to prevent progressive land salinization [31].

### 3.4. Vapor (Steam) Temperature Profile

The steam formed in each stage of the plant operation has its own specific temperature. This is essential in the model design in order to evaluate the design dimensions of the various stages. Nevertheless, it is known that this temperature is a function of the pressure of the steam inside the stages. As explained earlier, the temperature of the steam was evaluated by using the energy conservation equation and by assessing the latent heat of vaporization. This led to assessing the flashing vapor amount, which represents the mass flow of the steam, and hence, the enthalpy of the steam is specified. In this case, two characteristics are available in that the temperature of the steam can be directly found from the thermodynamic property tables. The calculated latent heat of vaporization was less than the calculated steam enthalpy, and this is quite the case when latent heat is the amount of heat (thermal energy) required to evaporate 1 kg of saturated liquid water and convert it into steam (vapor). According to Equations (28) and (29), the amount of energy released in each stage was assessed, and the temperature of the steam was also calculated, given that the enthalpy of the steam was already found.

The temperature of the steam decreased stage by stage from 89 °C to 53 °C. As shown in Table 3, it can be seen that the decrease in the steam temperature was not constant in the same way as the temperature drop of the brine water (feed water) as the liquid phase in each stage. This makes sense, in that the energy released was not constant for each stage. At the same time, the mass flow or steam flash was also not flashing in a constant manner. This indicates that the variation in the steam enthalpy also has the same aspect. It should be noted that the mass flow of desalinated water did not accumulate at a constant rate, given that it was totally dependent on the pressure drop per stage, as discussed later. To explain Table 3 below, first, the mass flow of the distillate water was already calculated (which was assumed to be equal to that of evaporated steam), and the energy released was calculated using Equation (28). By taking the calculated value of the steam enthalpy and applying Equation (29), by iteration, the steam temperature for each stage was assessed.

### 3.5. Energy Release and Pressure Drop

The pressure drop per stage or the pressure difference between the brine water streams in and out of each stage is the driving force with regard to vapor flashing. The temperature difference of the brine water streams (in and out) in a stage was the tracked function to evaluate the pressure drop. Starting from the first stage, the temperature of the brine water (before entering the first stage) was 93 °C, which is, at the same time, the top brine temperature. ∆T for the brine water stream at each stage had already been found (2.34 °C); thus, the temperature of the brine stream coming out of the first stage was around 90.5 °C. The pressure of the brine streams in and out for the first stage was 1.15 bar and 0.71 bar, respectively. If these values are checked against the thermodynamic property table, it can be seen that with these pressure values, the brine water remained in its liquid phase and released the amount of heat needed for vaporization. Note that the temperature profile of the whole brine water stream through the 16 stages was already found in the design phase. Thus, the pressure drop was calibrated to match the temperature of the brine water between the simulation results and the design results. Furthermore, in the design phase, the vapor mass flow (steam flash) was also found. Therefore, $m_{Bi}h_{Bi}-m_{Bo}h_{Bo}$ was verified, and the amount of thermal energy released was evaluated.

**Figure 14:**
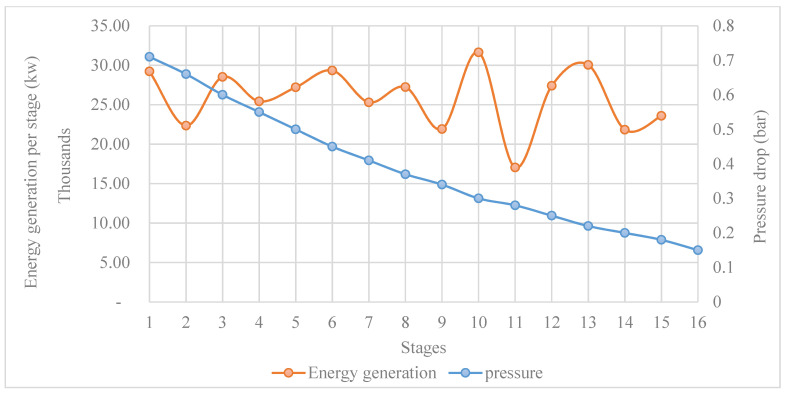
Variation in energy released per stage as a function of the pressure drop.

To interpret the variation in energy generation per stage, there is a need to return to the design phase once more. The latent heat of vaporization was found by taking the average temperature for all stages of the facility; its value was found to be around 2320 KJ/kg. In the first stage, around 11 kg/s of steam was vaporized and converted into free salt water. As can be seen in Figure 14, the amount of energy released was 29,211 KW, which is more than the energy required for vaporization. If a constant rate of mass flow of steam is vaporized (10 kg/s for example), in this case, around 23,200 KW of energy is required as latent heat of vaporization. At the same time, for example, in the second stage, the brine water streams flowing in and out have the parameters shown in Table 4.

By taking the difference between the energy in and out, it can be found that this stream released just 22,963 KW, while the required energy or latent heat of vaporization needed to vaporize 11 kg/s is around 23,200 KW. In this case, this stage will suffer from fatigue stress, and steam will not be formed. Consequently, the mass flow of steam vaporization decreases to around 8.4 kg/s to match the amount of energy required for vaporization. In this case, it will be 19,488 KW (8.4 × 2320). Note that the mass flow rate of brine water out of the second stage will be 2480 kg/s instead of 2479 kg/s, and the energy released will be 22,608 KW, as shown in Figure 14. This was the main cause of the fluctuating behavior in terms of energy generation for the various stages and the pressure drop. The pressure in the stages decreased from 0.71 bar to 0.15 bar in the last stage, while the pressure steps or difference between the in- and outflowing brine stream was 0.06 bar maximum and 0.02 bar minimum.

### 3.6. Effect of Temperature Variation on Water Intake

OT technology is influenced by seasonal temperature changes of the water at the intake source, as temperature changes directly affect the performance ratio of the desalination plant. When the seawater temperature drops to as low as 15 °C during the winter, operating under these conditions will result in a drastic reduction in the system’s performance ratio or in its production capability. A thermal performance ratio of 3 has been stated for MSF-OT units in winter conditions [22,32].

In this research, a preheater was inserted at the influent stream of the feed water. This was used to calibrate and increase the feed water temperature before entering the plant, where the brine water stream exiting from the last stage (stage 16) acts as a thermal energy source for the feed water. The effect of seawater (water intake) temperature is directly connected to the performance ratio, which represents an indicator of the desalination capacity of the desalination facility. Table 5 presents the effect of temperature change with regard to seawater on the free saltwater formation from the plant, where 30 °C was taken as the standard seawater temperature for the simulation, and the temperature effluent of the brine water from the last stage (stage 16) was kept at around 55 °C. This brine water stream is used in the preheater to moderate the temperature of the seawater before entering the plant, as can be seen in Table 5, which shows that the temperature of the seawater was 30 °C and increased to 48 °C when passing through the preheater. The temperature of the seawater decreased from 30 °C to 5 °C in 5 °C decrements. For each temperature value, the simulation in Ebsilon was performed to scale up the mass flow rate of the seawater required in such a way as to match the calibrated pressure profile of the desalination stages and to conserve the mass flow of the steam in the brine heater at its specified value (29.5 kg/s). The results show that the desalination capacity was reduced from 13,997 m^3^/day at 30 °C to 6,791 m^3^/day at 5 °C. The performance ratio consequently decreased from 5.49 at 30 °C to 2.66 at 5 °C. Nevertheless, the effect of temperature variation with regard to the seawater intake will affect the economic analysis of the whole proposed system.

## 4. Discussion

Thermal water desalination plays a major role in providing a secure source of water for many societies around the world. Multi-stage flash (MSF) occupies 46% of the desalination market in GCC countries [33,34]. At the same time, this technology needs a large amount of thermal energy to be delivered, as mentioned before [35]. For this reason, many researchers have highlighted the concept of using thermal energy produced from waste incinerators.

Dajnak et al. [36] concluded that the concept of Waste-to-Energy-to-Water needs further study to optimize the conversion route and to evaluate the economy of the concept relative to competing desalination energy sources. Thabit et al. [37] simulated a comprehensive waste incineration plant case study in Jordan for power generation and water desalination. They found that this concept increases the affordability of the whole system.

Pirotta et al. [38] examined the potential of energy retrieval from the MSW of Maltese for power generation and water desalination. The best scenario considered corresponds to a potential electric power of 10 MW or to a maximum of 4.8 million m^3^/year of desalinated water. It was concluded that incineration has the highest potential to maximize profits due to the optimal combination of heat production and electricity generation.

Mohammed et al. [39] utilized waste gases that emerged from oil refineries rather than burning them in the air. Hybrid MSF-MED (Multi-Effect Desalination) thermal desalination processes were utilized in this study to produce a total range of 100–40,000 m^3^/day. Ishaq et al.’s [40] trigeneration system for electricity, hydrogen and freshwater production using waste heat from a glass melting kiln was explained in their work.

At the same time, many investigations have been conducted to optimize MSF technology. Abdel Nasser et al. [41] reported a novel integrated MSF–MED thermal desalination technology and its techno-economics as compared to conventional systems such as brine recirculation MSF, MED–Thermal Vapor Compression, once-through MSF and plain MED. Ghaizza et al. [42] outlined how to correctly consider the overall energy consumption for a thermal plant with either MED or MED–Thermal Vapor Compression or MSF and demonstrated that an advanced internal geometrical design of an MSF type A can allow a large number of stages, enabling very high PR ≥ 12 to be reached, which gives comparable or even better results than MED. Hanshik Ch. et al. [43] studied the effect of increasing the top brine temperature on the performance of an existing MSF facility. The results indicated that the performance of the multi-stage evaporating system can be increased with the elevation of the top brine temperature. Bandi Ch. et al. [44] outlined the best mathematical model with the differential evolution algorithm. They proved that the model was the most effective one to generate feasible optimal design variable values for MSF-BR and MSF-M but not for MSF-OT processes.

However, the presented work has many advantages, as it (1) introduces a systematic model to design a once-through multi-stage flash plant, (2) predicts the performance of the stages in terms of the temperature drop of the brine flow stream at the lower section of the stages and temperature increment of the feed water at the upper section (condensers) stage by stage, (3) precisely evaluates the amount of thermal energy required to operate the plant by the brine heater, (4) assesses the mass flow rate of the steam needed to be injected into the brine heater according to the assumed conditions, (5) evaluates the NEA parameter, which is the key to specifying the energy content (enthalpy) of the vapor in each stage and its temperature, (6) inserted the designed variables and parameters in Ebsilon software to simulate 16 stages of OT-MSF and provides all outcomes, as presented in the Results and Discussion section, (7) simulated the effect of the seawater temperature change on the performance ratio of the destination plant and (8) concludes that the source of thermal energy required to drive the desalination plant can be supplied by a hybrid system consisting of waste incineration–parabolic trough solar field.

On the other hand, there are some drawbacks: (1) water distillate temperature on the demister itself was neglected according to the literature to facilitate the model and simulation process; (2) OT-MSF was considered in this article rather than brine recirculation (BR), where the latter utilizes less seawater for the same amount of freshwater produced: it should be noted that BR produces a high salt concentration in brine discharge (for more differences between OT and BR techniques, please see [19]); and (3) the temperature drop of brine and the temperature increment of feed water in the condenser tube bundle were considered constant.

## 5. Conclusions

A comprehensive database and experience have been built from more than 2000 MSF plants in the MENA region. It is for this reason that this technology was considered in this research. The Combined Heat Power (CHP) cycle can operate MSF facilities. This classical operational procedure was embedded in this research to connect the hybrid power plant (waste incineration–parabolic trough) to the MSF facility. The main purpose of this connection is to increase the affordability of the whole power plant in terms of economic aspects and decrease the heat loss from the plant by adding an efficient usage of thermal energy. The results in this paper show that erecting an MSF facility within a hybrid system can be implemented by harvesting around 69,330 KW of thermal energy in the form of steam by transferring it into the feed water in a brine heater. Steam can be supplied for a desalination plant by drawing a steam line from the steam turbine series (specifically from the second steam turbine) to supply the required thermal energy to operate the 16 stages of the MSF plant. It can be concluded that there are two main factors affecting the performance of the desalination process: seawater temperature and salt concentration. In this study, the latter was assumed to be constant at 42,000 mg/L, while ranges in the temperature of the seawater from 30 °C to 5 °C were considered. The temperature drop required us to reduce the mass flow of seawater in order to conserve the amount of thermal energy needed to be supplied to the MSF plant from the power plant through the steam line into the brine heater. In other words, if the same amount of seawater is desalinated despite a drop in temperature, this will increase the mass flow of the drawn steam flow from the power plant. This means that a drop in the power generation of the power plant will occur, or more MSW will need to be loaded into the furnace. This could lead to the overload of the designed capacity of the incineration plant, thereby increasing the cost of the incineration components and flue gas purification. On the other hand, the temperature variation in the seawater will affect the loading rate of the MSW of the incineration plant, which will need to be continuously calibrated. It is more convenient to decrease the mass flow of seawater that needs to be desalinated when its temperature decreases, leading to a proportional decrease in the performance ratio, as shown before. The effect of the temperature drop on the part of the seawater extends to the financial aspects of the entire system, in that the income of the plant will also decrease as the performance ratio decreases. Furthermore, it can be concluded that for each 5 °C drop in the seawater temperature, the MSF plant water production will be reduced by 1000–3000 m^3^/day.

The design phase can be verified in the simulation process and in reality, with a small deviation in the temperature of the brine stream (the feed water), which will decrease at a constant rate of around 2.34 °C/stage. At the same time, the condenser stream (feed water in the condenser zone) temperature was increased at the same rate. The pressure drop in the brine zone has a main role to play in specifying the latent heat of vaporization. The pressure dropped from 0.71 bar in the first stage to 0.15 bar in the last stage, while the steam energy content (or latent heat of vaporization) decreased from 2660 KJ/kg to 2598 KJ/kg, respectively.

Variation in the distillate water mass flow was recognized from stage to stage. This is very important to keep in mind in order to prevent the incidence of fatigue stress in a stage. At 75 °C, saturated liquid water needs around 2320 KJ/kg of thermal energy to be converted into saturated steam. Consequently, if this amount of energy is not provided, fatigue stress will appear in a stage, and it will not be possible to desalinate more free saltwater. Therefore, a constant mass flow rate of distillate water coming out of each stage is not possible due to the variation in mass flow and in the pressure of the brine stream from stage to stage.

## Figures and Tables

**Table 1:** Technical design parameters used in the model.

Parameter	Unit	Symbol	Value	Reference of Values
Feed water flow rate	Kg/s	M_f_	2500	Assumed
Salt concentration	Mg/L	X_i_	42,000	Literature
Feed seawater temperature	°C	T_f_	48	Calibrated
Steam temperature	°C	T_s_	130	Calibrated
Top brine temperature	°C	T_0_	93	Calibrated
Brine temperature in the last stage	°C	T_n_	54	Calibrated
Heat capacity of liquid streams	kJ/kg °C	C_p_	4.18	Literature
Vapor velocity in the last stage	m/s	V_v_	6	Literature
Brine mass flow rate per width	Kg/ms	V_b_	180	Literature
Weir friction coefficient	C_d_	-	0.5	Literature
Number of stages	n	-	16	Designed

**Table 2:** Key differences between the main desalination technologies (MSF, MED and RO) in terms of unit capacity and distillate salinity. TDS: Total Dissolved Salt; ppm: part per million.

Technology	MSF	MED	RO
Typical unit size (m^3^/day)	50,000–70,000	5000–15,000	24,000
Distillate quality—TDS (ppm)	≈10	≈10	<500

**Table 3:** Evaluated steam temperature by iteration using Equations (28) and (29).

Stage	$m_{Bi}h_{Bi}-m_{Bo}h_{Bo}=m_{st}h_{st}$ Equation (28) (kW)	$h_{st}=\;2500.15+1.947036\;T-0.00195\;T^{2}$ Equation (29) (KJ/kg)	Steam Temperature °C	Distillate Mass Flow (kg/s)	Steam Enthalpy (KJ/kg)
1	29,211.60	2659.11	89.66	10.98	2660.44
2	22,339.80	2655.71	87.58	19.38	2657.29
3	28,519.49	2651.72	85.10	30.13	2653.22
4	25,425.54	2648.16	82.90	39.7	2649.60
5	27,205.90	2644.25	80.50	50	2645.72
6	29,333.77	2639.99	77.90	61.12	2641.25
7	25,297.72	2636.20	75.60	70.7	2637.38
8	27,228.23	2632.06	73.10	81	2633.29
9	21,918.95	2629.39	71.20	89.4	2631.33
10	31,634.06	2623.87	68.20	101.4	2624.80
11	17,036.07	2621.00	66.70	107.9	2622.55
12	27,389.71	2616.94	64.10	118.4	2617.77
13	30,040.06	2612.00	61.20	129.8	2612.86
14	21,829.62	2608.24	59.00	138.3	2609.33
15	23,589.30	2604.62	56.90	147.3	2605.40
16	39,534.78	2597.87	53.00	162.5	2598.58

**Table 4:** Brine stream properties before and after stage 2, with 10 kg/s flash steam assumed.

Stage 2		
Parameter	Brine Water in	Brine Water out
Pressure (Bar)	0.71	0.66
Temperature (°C)	90.5	88.6
Mass flow (kg/s)	2489 (take off 10 kg/s as vaporized)	2479
Enthalpy (KJ/kg)	362.7	354.9

**Table 5:** Effect of seawater temperature change on the performance of water desalination plant.

Temperature of Sea Water Intake °C	Mass Flow of Water Intake (kg/s)	Mass Flow of Desalinated Water (kg/s)	Performance Ratio	Temperature out of the Preheater °C	Desalinated Water m^3^/day
30	2500	162	5.49	48	13,997
25	2000	126	4.27	47	10,886
20	1800	113	3.83	45.8	9763
15	1500	94	3.19	44	8122
10	1400	88	2.98	43	7603
5	1250	78.6	2.66	41.7	6791
